# Feedstock/pretreatment screening for bioconversion of sugar and lignin streams via deacetylated disc-refining

**DOI:** 10.1186/s13068-024-02492-7

**Published:** 2024-04-05

**Authors:** Darren J. Peterson, Changyub Paek, Ling Tao, Ryan Davis, Xiaowen Chen, Roman Brunecky, Matthew Fowler, Richard Elander

**Affiliations:** 1 National Renewable Energy Laboratory, 15013 Denver West Pkwy, Golden, CO 80401 USA; 2 ExxonMobil Technology and Engineering Company, 1545 US 22 E, Annandale, NJ 08801 USA

## Abstract

Recent publications have shown the benefits of deacetylation disc-refining (DDR) as a pretreatment process to deconstruct biomass into sugars and lignin residues. Major advantages of DDR pretreatment over steam and dilute acid pretreatment are the removal of acetyl and lignin during deacetylation. DDR does not generate hydroxymethylfurfural (HMF) and furfural which are commonly produced from steam and dilute acid pretreatments. Acetate, lignin, HMF, and furfural are known inhibitors during enzymatic hydrolysis and fermentation. Another advantage of deacetylation is the production of lignin-rich black liquor, which can be upgraded to other bioproducts. Furthermore, due to the lack of sugar degradation during deacetylation, DDR has significantly less sugar loss than other pretreatment methods. Previous studies for DDR have primarily focused on corn stover, but lacked the investigative studies of other feedstocks. This study was designed to screen various DDR process conditions at pilot scale using three different feedstocks, including corn stover, poplar, and switchgrass. The impact of the pretreatment conditions was evaluated by testing hydrolysates for bioconversion to 2,3-butanediol. Pretreatment of biomass by DDR showed high-conversion-yields and 2,3-BDO fermentation production yields. Techno-economic analysis (TEA) of the pretreatment for biomass to sugar was also developed based on NREL’s Aspen Model. This study shows that the cellulose and hemicellulose in poplar was more recalcitrant than herbaceous feedstocks which ultimately drove up the sugar cost. Switchgrass was also more recalcitrant than corn stover but less than poplar.

## Background

Lignocellulosic biomass conversions to fuels and chemicals continue to undergo extensive research as an alternative to traditional fossil-oil-based fuels and chemicals. In 2012, the National Renewable Energy Laboratory (NREL) completed an extensive study illustrating the state of technology and techno-economics of steam, dilute acid, and deacetylated dilute acid pretreatments of corn stover [1, 2]. The technical report lays out results using deacetylation, dilute acid pretreatment, enzymatic hydrolysis, and ethanol fermentation using a strain of *Zymomonas mobilis* developed at NREL. Dilute acid pretreatment of corn stover produces acetate, hydroxymethylfurfural (HMF), and furfural which are inhibitory to enzymatic hydrolyses and fermentations [3, 4]. Because HMF and furfural are degradation products from C5 and C6 sugars, the loss of sugars in dilute acid pretreatment is significant. Because DDR does not produce HMF or furfural, the sugar loss is significantly lower than other pretreatment methods. The remaining lignin in the insoluble solids can also inhibit enzymatic hydrolysis [2, 3]. While deacetylation with sodium hydroxide prior to dilute acid pretreatment is sufficient for removing acetate and lignin, it does not prevent the co-production of HMF nor furfural downstream during dilute acid pretreatment [5]. Furthermore, acetylation provided a lignin-rich black liquor which can be separated and upgraded to other valuable chemicals [6]. Recent studies have shown that substituting dilute acid pretreatment with mechanical refining after deacetylation sufficiently exposes the cellulose and hemicellulose for high solids enzymatic hydrolysis [7–10]. These studies have also shown higher sugar yields from enzymatic hydrolysis as well as higher fermentation yields [5, 7–9]. While enzymatic hydrolysis and fermentation yields after deacetylation disc-refining (DDR) pretreatment have been investigated and found to be favorable for corn stover, other promising feedstocks have not been investigated in as much depth, such as poplar and switchgrass.

To date, NREL has focused DDR pretreatment studies primarily around corn stover. However, poplar and switchgrass feedstocks have shown promising results via steam and dilute acid pretreatments [11, 12]. In light of such favorable findings for these alternative feedstocks across other pretreatment options, it is worth exploring their potential as viable DDR pretreatment candidates. Furthermore, given the dearth of data for poplar and switchgrass processing via DDR pretreatment, comparable techno-economic analysis (TEA) on such a dataset has not been made available, though TEA assessment for deacetylation mechanical refining (DMR), similar to DDR, of corn stover biomass has been conducted [13].

**Fig. 1:**
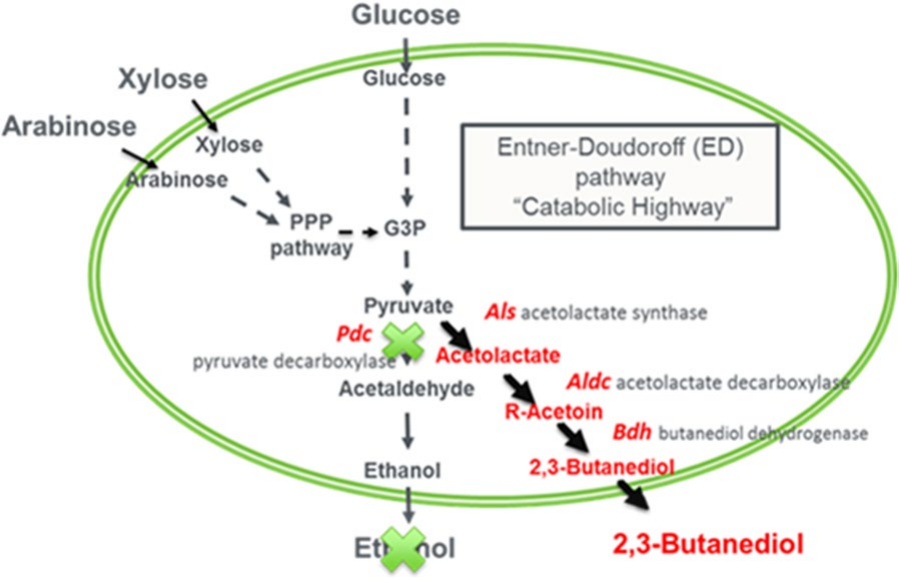
Metabolic pathway deletions and insertions for producing 2,3-BDO for C5/C6 sugars (xylose and glucose) in the Z. mobilis YC1 strain

The goal of this study was to evaluate the differences in biomass deconstruction to monomeric sugars as well as fermentation of those sugars in hydrolysates to a biological intermediate product of interest as a means of understanding the effect of potential inhibitory compounds in the hydrolysates from different DDR pretreatment process conditions. NREL’s existing *Z. mobilis* organism (YC1 strain) was chosen as a representative organism to use for the hydrolysate fermentability testing. This strain was originally developed to produce ethanol from mixed C5/C6 biomass-derived sugars, xylose and glucose, and has been modified to knock out the ethanol pathway and engineered to produce 2,3-butanediol (2,3-BDO). 2,3-BDO is a major platform chemical of interest, as 2,3-BDO can be catalytically upgraded to produce diesel and jet fuel range molecules as well as large-volume chemical products (e.g., butadiene). The metabolic pathway deletions and insertions for producing 2,3-BDO are shown in Fig. 1. The particular YC1 strain used has the capabilities to utilize glucose and xylose, but not arabinose. Other related strains that are in early-stage development do have the ability to utilize arabinose, but they were not used in this study [14].

The TEA model developed by NREL as utilized in this study focuses on production of lignocellulosic sugars through pretreatment and enzymatic hydrolysis. The TEA model incorporates experimental data to provide a direct comparison of feedstocks and DDR pretreatment conditions on resulting economics of sugar production.

## Results

### Deacetylation

**Fig. 2:**
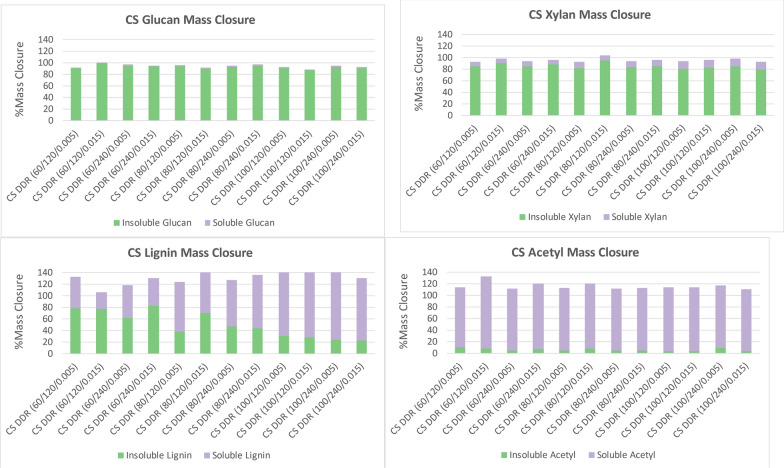
Corn stover (CS) deacetylation mass closure of glucan (top left), xylan (top right), lignin (bottom left), and acetate (bottom right). Lignin conversion yield to black liquor is likely overestimated due to the lack of an accurate quantifying method. Acetyl conversion yield is high due to under quantifying acetyl in the raw feedstock. Acetyl is nearly all removed from the solids fraction. Lignin is removed more when the concentration of sodium hydroxide is increased. Most of the glucan and xylan, remain within the insoluble solids fraction. X/Y/Z represents NaOH conc (g/kg)/residence time (min)/disc-refining gap (in.)

Figure 2 displays the mass closure of corn stover for glucose, xylose, lignin, and acetyl after deacetylation. The mass closure is separated by structural fraction that remained in the insoluble solids fraction of the caustic slurry and the soluble fraction that was dissolved during deacetylation. The sample identification follows the following nomenclature: DDR—deacetylation disc-refined pretreatment; CS—corn stover; 60, 80, and 100–concentration of sodium hydroxide in grams of sodium hydroxide per kilograms of dry biomass; 120 and 240—residence time in minutes; and 0.005 and 0.015—disc gap in the disc-refiner measured in inches. Only glucose and xylose are displayed as they are the primary fermentable sugars of interest. Soluble lignin carries a higher degree of uncertainty and is likely overestimated as reported due to a lack of an accurate quantifying method for black liquor. Acetyl mass closure is also over 100% due to the under quantifying acetyl in raw feedstock. The glucan remained in the insoluble solids fraction, showing that cellulose was largely unaffected by deacetylation. The xylan was slightly solubilized during deacetylation. It is common for a small fraction of hemicellulose to solubilize during deacetylation. Fortunately, most of the hemicellulose was not lost during the deacetylation process. As expected, the lignin solubility increased as the sodium hydroxide concentration increased. Over 90% of the acetyl was removed from the insoluble solids fraction. The removal of acetate is crucial to prevent inhibition of enzymatic hydrolyses and fermentations. The residence time did not appear to have significant impact on solubility.

**Fig. 3:**
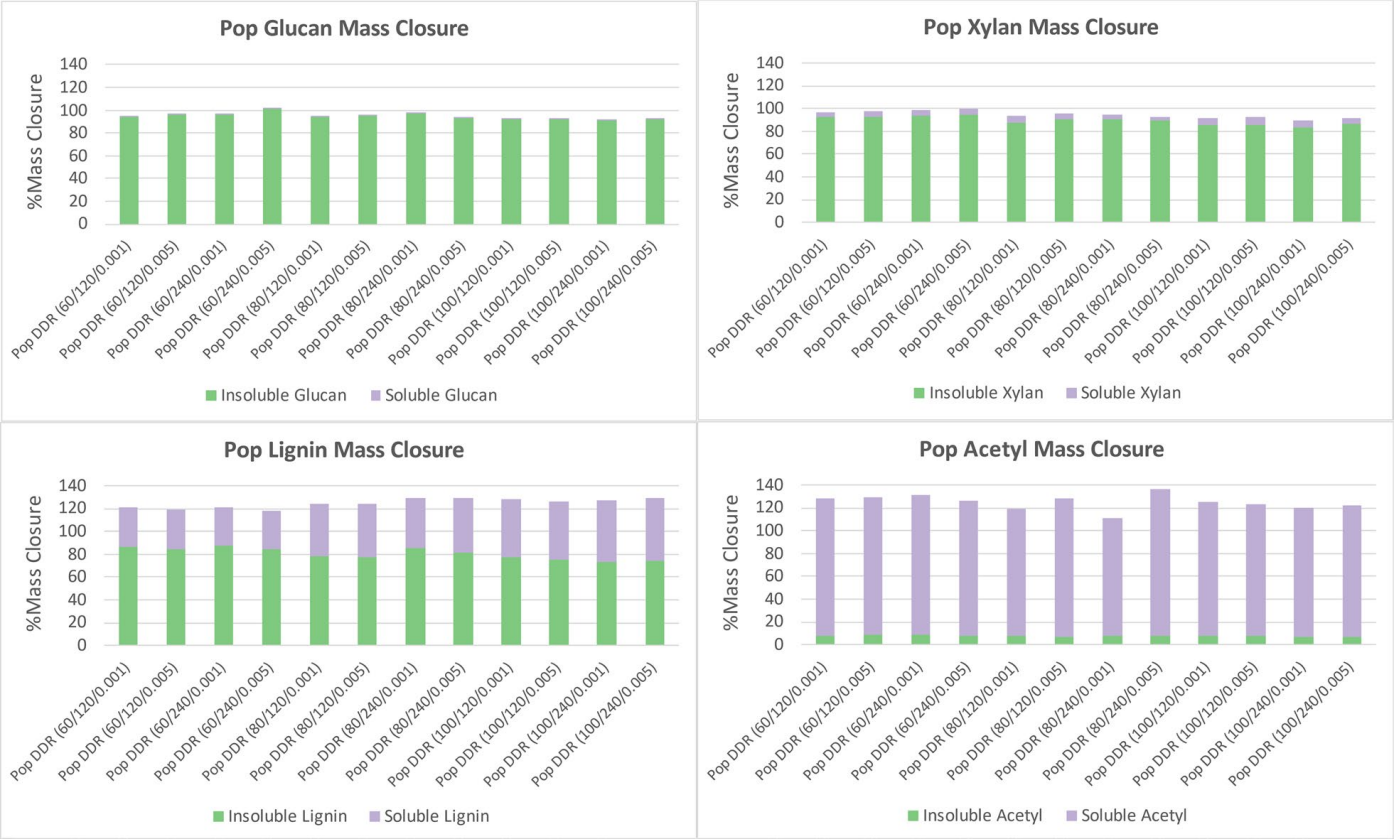
Poplar (Pop) deacetylation mass closure of glucan (top left), xylan (top right), lignin (bottom left), and acetyl (bottom right). Lignin conversion yield to black liquor was likely overestimated due to the lack of an accurate quantifying method. Acetyl conversion yield was high due to under quantifying acetyl in the raw feedstock. The acetyl was nearly all removed from the insoluble solids fraction. Lignin was removed more when the concentration of sodium hydroxide was increased, but significantly less than corn stover. Most of the glucan and xylan, remain with the insoluble solids fraction. X/Y/Z represents NaOH conc (g/kg)/residence time (min)/disc-refining gap (in.)

Figure 3 displays the mass closure of poplar for glucose, xylose, lignin, and acetyl after deacetylation in the same manner that corn stover was displayed. The nomenclature is the same with Pop representing Poplar and the gap space for disc-refining changing from 0.001 to 0.005. The glucan and xylan in poplar followed similar trends observed in corn stover, where very little of the cellulose and hemicellulose were solubilized during deacetylation. The acetyl was also removed near completion. However, the lignin was not solubilized as well for poplar as it was for corn stover. Over half the lignin remained with the insoluble solids fraction. The solubility did increase as the sodium hydroxide concentration increased. Further solubility could likely be achieved with an increase in sodium hydroxide. However, increasing sodium hydroxide has a negative environmental impacts [13] and therefore, were not investigated in this project [15]. The increase in residence time had minimal impact on the solubility of poplar.

**Fig. 4:**
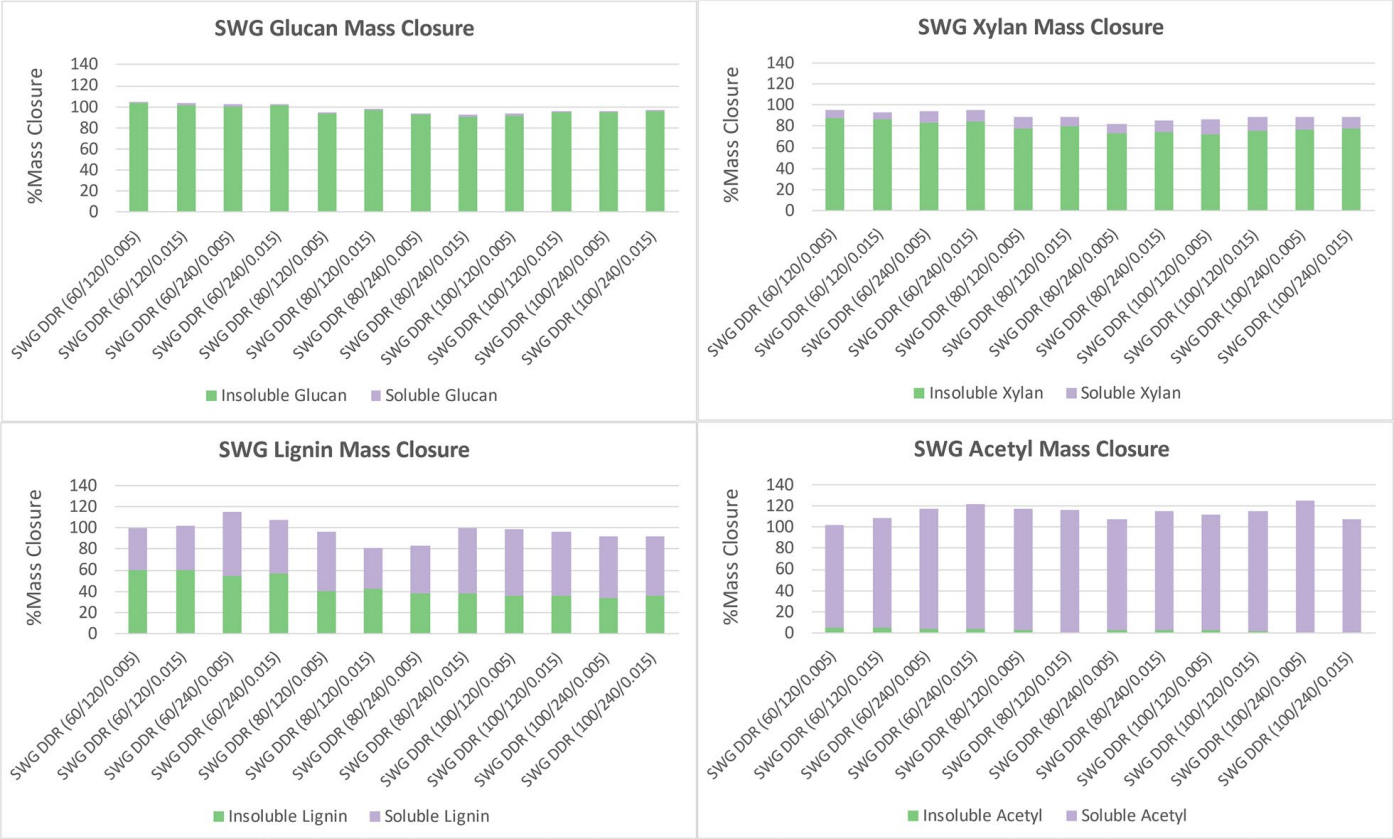
Switchgrass (SWG) deacetylation mass closure of glucan (top left), xylan (top right), lignin (bottom left), and acetyl (bottom right). Lignin conversion yield to black liquor was likely overestimated due to the lack of an accurate quantifying method. Acetyl conversion yield was high due to under quantifying acetyl in the raw feedstock. The acetyl is nearly all removed from the insoluble solids fraction. Lignin is removed more when the concentration of sodium hydroxide is increased. Lignin was removed more efficiently with switchgrass than poplar but not as well as with corn stover. The glucan remained with the insoluble solids fraction with the xylan increasing as the concentration of sodium hydroxide increased. X/Y/Z represents NaOH conc (g/kg)/residence time (min)/disc-refining gap (in.)

Figure 4 displays the mass closure of switchgrass for glucose, xylose, lignin, and acetyl after deacetylation in the same manner as corn stover and poplar. The nomenclature is the same as corn stover with SWG representing Switchgrass. The glucan in switchgrass followed similar trends observed in corn stover and poplar, where very little cellulose was solubilized during deacetylation. The xylan in switchgrass did have a slight increase in solubility as the concentration of sodium hydroxide increased with a maximum of 14%. The acetyl was also removed near completion. The lignin in switchgrass did increase solubility as the sodium hydroxide concentration increased. Overall, the lignin from switchgrass had a higher solubility in switchgrass than poplar, though lower than that observed with corn stover. The residence time had minimal impact on solubilizing switchgrass, much like poplar and corn stover.

### Disc-refining

**Fig. 5:**
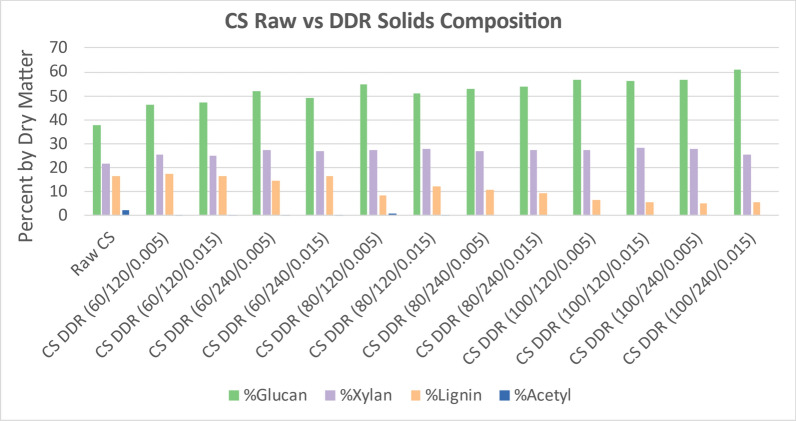
DDR corn stover solids (CS) composition compared to raw corn stover. Following the same trend as was observed with just deacetylated corn stover, the acetyl composition is minimal while the lignin composition decreases with the increase of sodium hydroxide. Xylan composition remains consistent throughout the severity curve whereas glucan increases with severity due to the loss of lignin. The residence time and gap space for disc-refining does not appear to affect the overall composition of DDR corn stover. X/Y/Z represents NaOH conc (g/kg)/residence time (min)/disc-refining gap (in.)

Figure 5 displays the change in structural composition components of interest of the DDR corn stover as compared to raw corn stover. Because deacetylation acts as an extraction step and an excess of water is used during disc-refining, the extractive data were excluded from Fig. 5. Because a high fraction of the lignin and acetyl was removed during deacetylation, the proportional fraction of solids has a large increase in glucan and xylan as the sodium hydroxide concentration increases. As observed previously, the residence time did not affect the solids composition. The gap space for disc-refining did not affect the solids composition.

**Fig. 6:**
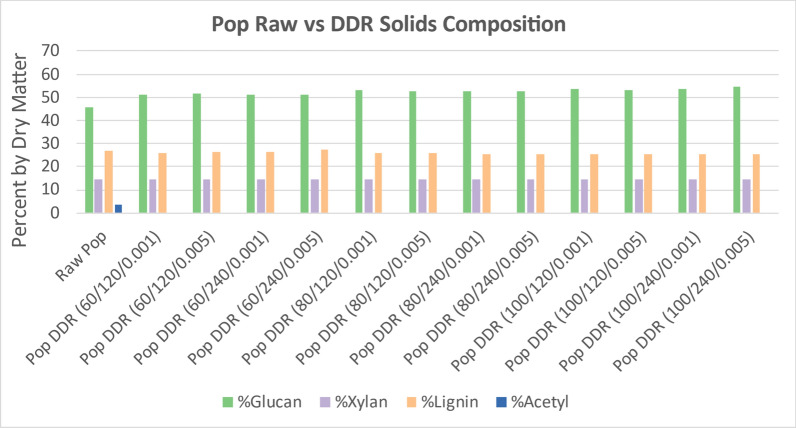
DDR poplar (Pop) solids composition compared to raw poplar. Following the same trend as was observed with just deacetylated poplar, the acetyl composition is minimal while the glucan, xylan, and lignin compositions remained consistent as deacetylation severity increased. Nearly all the acetyl was removed. X/Y/Z represents NaOH conc (g/kg)/residence time (min)/disc-refining gap (in.)

Figure 6 displays the change in structural composition components of interest of the DDR poplar as compared to raw poplar. Because a smaller fraction of the lignin was removed during deacetylation than corn stover, the proportional fraction of solids has a smaller increase in glucan and xylan. Furthermore, the proportional glucan and xylan in the solids remains consistent as the sodium hydroxide concentration increased. The proportional lignin in the DDR poplar solids also remains consistent with the increase in sodium hydroxide. As observed previously, the residence time did not affect the solids composition. The gap space for disc-refining did not affect the solids composition.

**Fig. 7:**
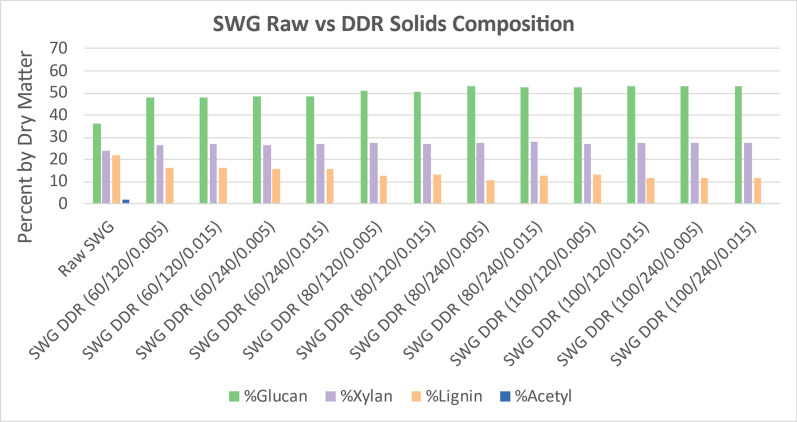
DDR switchgrass (SWG) solids composition compared to raw switchgrass. Switchgrass showed a high proportional fraction of glucan and xylan as the sodium hydroxide concentration was increased unlike poplar but similar to corn stover. However, not as much lignin was removed from switchgrass as was removed from corn stover, therefore, the increase of glucan and xylan was not as significant. X/Y/Z represents NaOH conc (g/kg)/residence time (min)/disc-refining gap (in.)

Figure 7 displays the change in structural composition components of interest of the DDR switchgrass as compared to raw switchgrass. The solids composition of DDR switchgrass followed the same trends as corn stover. While the lignin was removed from the switchgrass better than poplar, it was not removed as well as corn stover. This resulted in a proportional increase in glucan and xylan, but not as much as for corn stover.

### Enzymatic hydrolysis

**Fig. 8:**
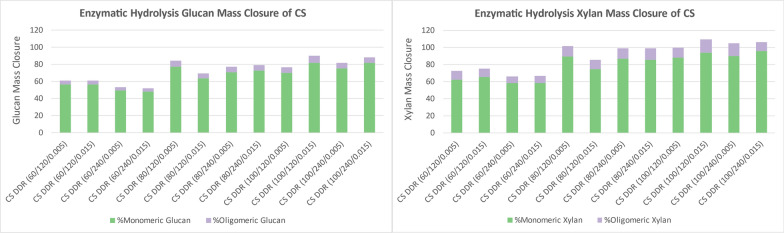
DDR corn stover (CS) enzymatic hydrolysis yields of glucose (left) and xylose (right). The glucan and xylan increased solubility as the sodium hydroxide concentration increased during deacetylation. The residence time during deacetylation might have a slight impact but not as much as sodium hydroxide concentrations. X/Y/Z represents NaOH conc (g/kg)/residence time (min)/disc-refining gap (in.)

The enzymatic hydrolysis yields in Fig. 8 show the glucan and xylan that were converted and solubilized into monomeric and oligomeric glucose and xylose from DDR corn stover. Monomeric sugars are preferred for subsequent processing through fermentation. Favorable fractions of glucan and xylan from DDR corn stover are solubilized and converted into monomeric sugars for all conditions, reaching 75% for glucan and 85% for xylan when the sodium hydroxide concentrations during deacetylation is 80 g/kg of dry biomass and higher, with the exception of 120 min residence time and 0.015-in. gap space. The residence time during deacetylation may improve the digestibility of the corn stover slightly, but not as much as the overall sodium hydroxide concentrations. The combination of shorter residence time and larger gap space at 80 g/kg of dry biomass may not have been severe enough and hindered the enzymatic hydrolysis when compared to other DDR conditions. The disc-refining was also done with a 12-in. disc-refiner which historically has not been as affective as a 36-in. disc-refiner [8].

**Fig. 9:**
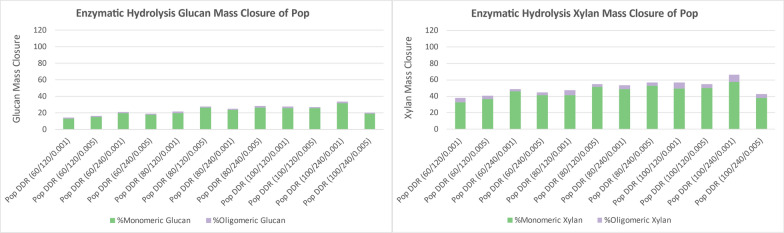
DDR poplar (Pop) enzymatic hydrolysis yields of glucose (left) and xylose (right). Both glucan and xylan in poplar had poor conversion for all DDR conditions. X/Y/Z represents NaOH conc (g/kg)/residence time (min)/disc-refining gap (in.)

The enzymatic hydrolysis yields in Fig. 9 show DDR poplar feedstock did not digest as well as corn stover across the severity conditions of DDR evaluated. Most of the glucan remained in the insoluble solids and the best xylan conversion was only 57%. The poor enzymatic hydrolysis yields for poplar are the result of DDR pretreatment not removing an adequate amount of the lignin and potential incompatibility with poplar.

**Fig. 10:**
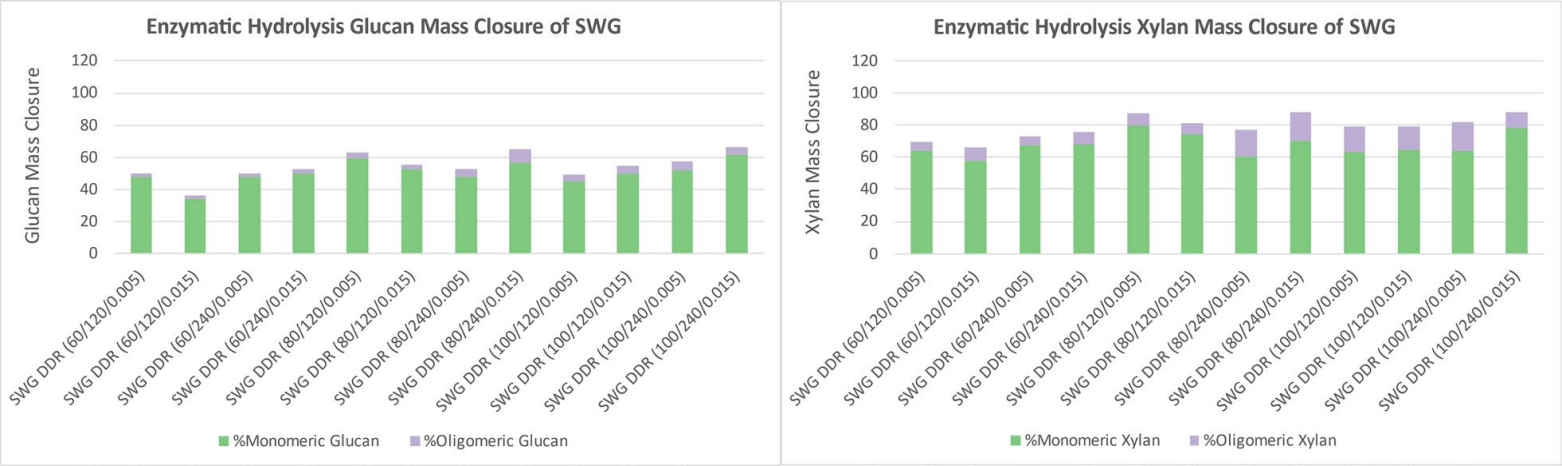
DDR switchgrass (SWG) enzymatic hydrolysis yields of glucose (left) and xylose (right). The glucan and xylan conversion for DDR switchgrass was more favorable than poplar but not as good as corn stover. X/Y/Z represents NaOH conc (g/kg)/residence time (min)/disc-refining gap (in.)

The enzymatic hydrolysis yields in Fig. 10 show the glucan and xylan that were converted and solubilized into monomeric and oligomeric glucose and xylose from DDR switchgrass. The glucan and xylan conversion for DDR switchgrass was not as favorable as corn stover but better than poplar. The lignin was not removed as efficiently from switchgrass during deacetylation as corn stover. The glucan maximum conversion maximum 60% and the maximum xylan conversion was 80%.

### Fermentation

**Fig. 11:**
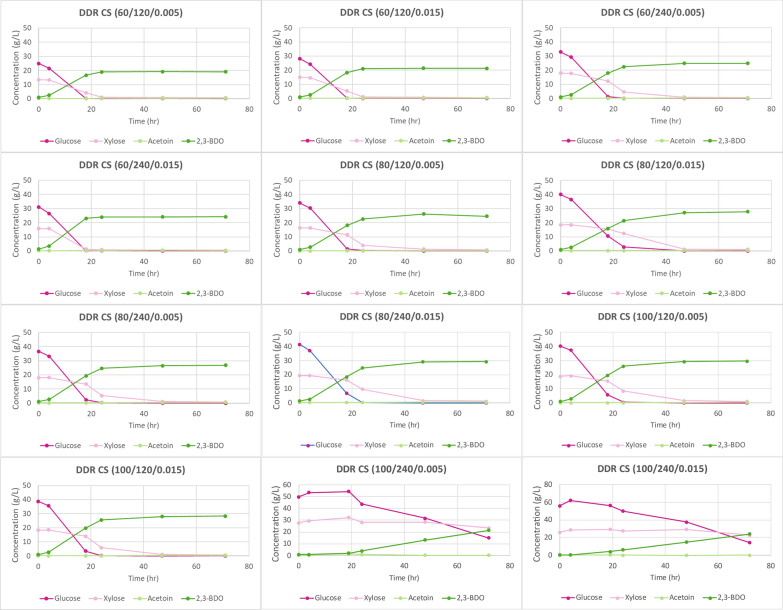
Time course data for DDR corn stover (CS) fermentation. All of the glucose and xylose are consumed and nearly all off the monomeric glucose and xylose are converted into fermentation products, 2,3-BDO and acetoin. X/Y/Z represents NaOH conc (g/kg)/residence time (min)/disc-refining gap (in.)

**Fig. 12:**
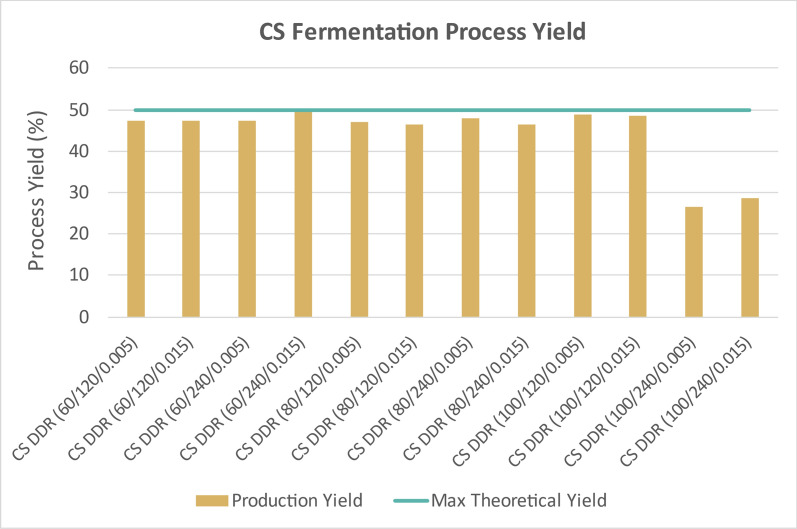
Fermentation process yields of DDR corn stover (CS). The maximum theoretical yield for glucose and xylose to 2,3-BDO and acetoin is 50%. All the glucose and xylose were consumed and the fermentation process yields were near the maximum, greater than 45%. The lack of inhibitors presents during fermentation allowed for high fermentation process yields. X/Y/Z represents NaOH conc (g/kg)/residence time (min)/disc-refining gap (in.)

Figure 11 displays the time course data for the DDR corn stover fermentations on corn stover hydrolysate. The time course data show that all the monomeric glucose and xylose were consumed except for the corn stover that was deacetylated with 100 g sodium hydroxide per kg of biomass for 240 min. The lack of inhibitors, namely lignin, acetate, HMF, and furfural allowed for complete sugar consumption, except for the two conditions previously mentioned. This was probably due to residual sodium hydroxide present during the fermentation. Figure 12 shows the fermentation process yields for DDR corn stover. The maximum theoretical process yields for glucose and xylose to 2,3-BDO and acetoin is 50%. All the fermentations for DDR corn stover were near the maximum theoretical yields, exceeding 45% except the two conditions discussed previously.

**Fig. 13:**
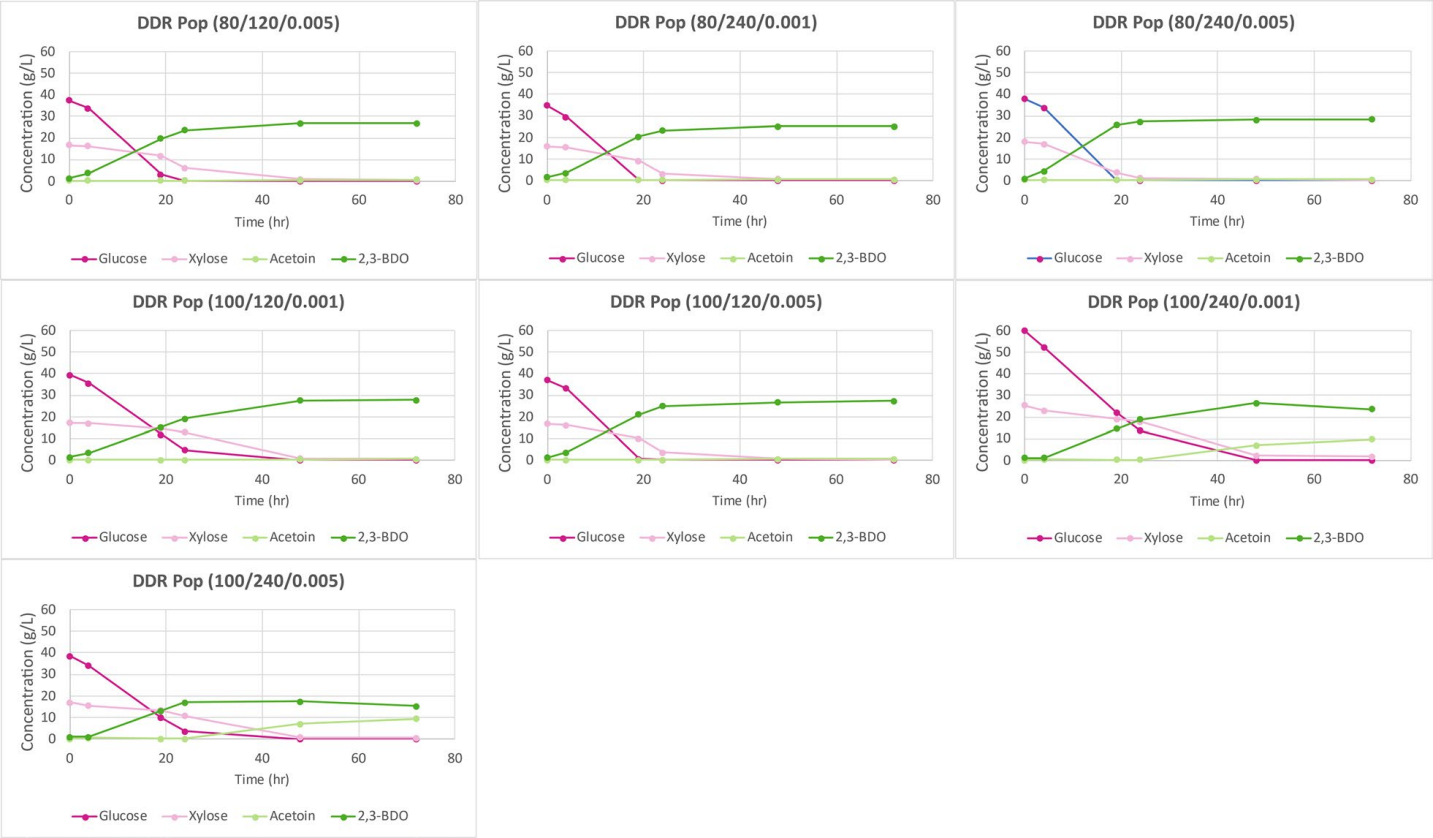
Time course data for DDR poplar (Pop) fermentation. DMR Pop (60/120/0.001), DMR Pop (60/120/0.005), DMR Pop (60/240/0.001), DMR Pop (60/240/0.005), and DMR Pop (80/120/0.001) were not fermented due to a lack of sufficient hydrolysate material. Like DDR corn stover, all of the available glucose and xylose were consumed. X/Y/Z represents NAOH conc (g/kg)/residence time (min)/disc-refining gap (in.)

**Fig. 14:**
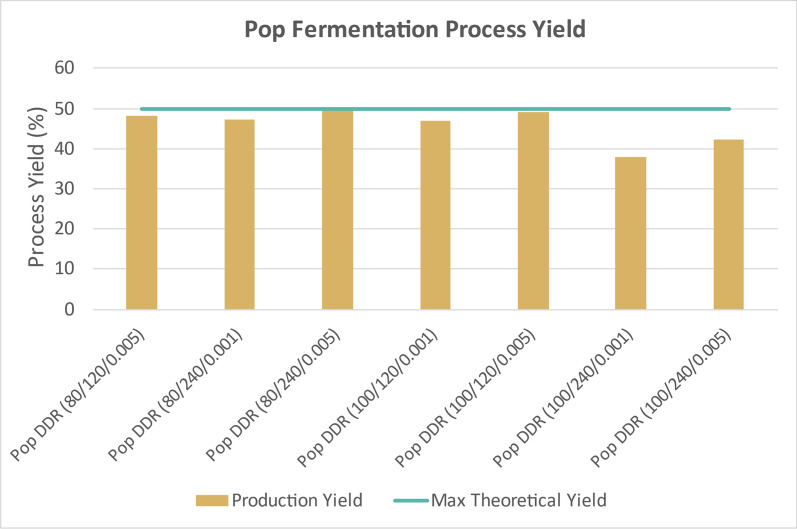
Fermentation process yields of DDR poplar (Pop). All the glucose and xylose were consumed and the fermentation process yields were near the maximum, greater than 45% except for Pop DDR (100/240/0.001) and Pop DDR (100/240/0.005) which ranged from 35–40%. The high fermentation process yields for DDR poplar are similar to DDR corn stover. X/Y/Z represents NaOH conc (g/kg)/residence time (min)/disc-refining gap (in.)

Figure 13 displays the time course data for the DDR poplar samples that underwent fermentations. Deacetylation of poplar with only 60 g/kg dry biomass of sodium hydroxide did not have enough liquor phase material after enzymatic hydrolysis to perform fermentation. DDR Poplar (80/120/0.001) did not produce enough liquor from enzymatic hydrolysis for fermentation either. This was due to poor enzymatic hydrolysis yields. The time course data show that all the available monomeric glucose and xylose were consumed much like DDR corn stover, again enabled by a general lack of inhibitors. Figure 14 shows the fermentation process yields for DDR poplar. The maximum theoretical process yields for glucose and xylose to 2,3-BDO and acetoin is 50%. Most of the fermentations for DDR poplar were near the maximum theoretical yields, exceeding 45% based on available monomeric sugars, much like DDR corn stover. However, the most severally pretreated DDR poplar, those at 100 g/kg of dry biomass of sodium hydroxide with a 240-min residence during deacetylation had a significantly lower process yield during fermentation, ranging from 35 to 40%.

**Fig. 15:**
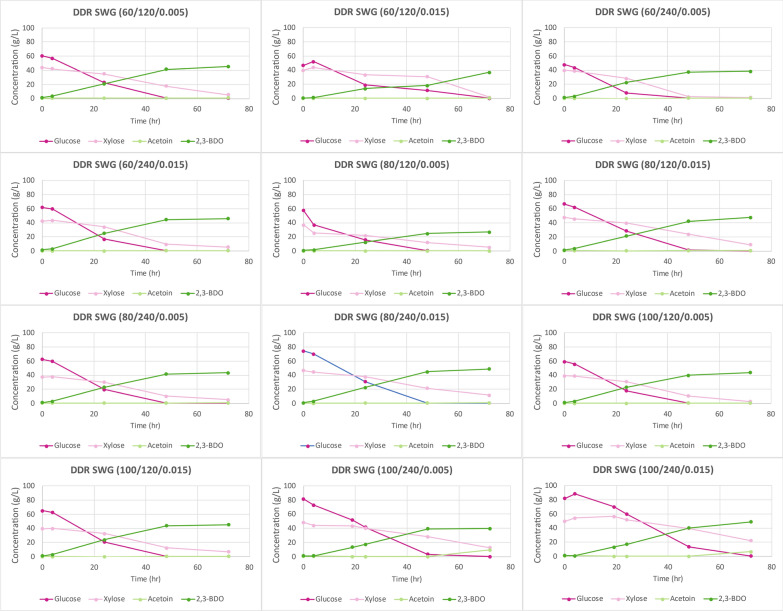
Time course data for DDR switchgrass (SWG) fermentations. Glucose was consumed some xylose left unconverted. Unconsumed xylose was not observed for DDR corn stover nor DDR poplar. X/Y/Z represents NaOH conc (g/kg)/residence time (min)/disc-refining gap (in.)

**Fig. 16:**
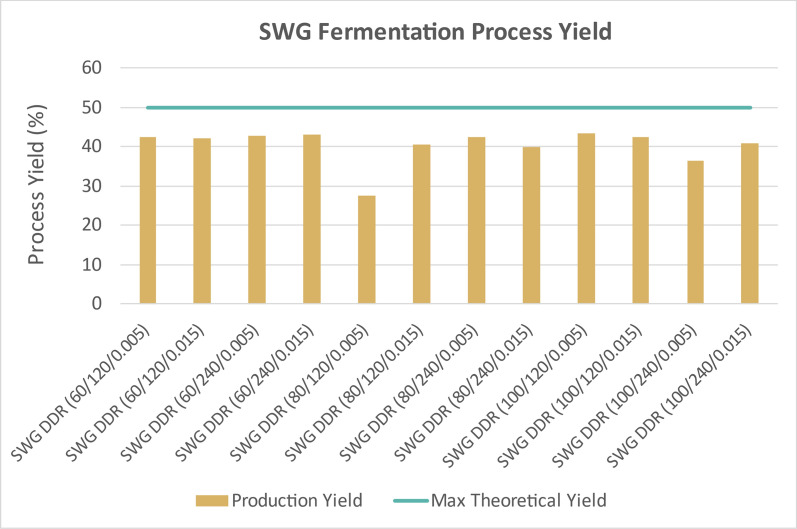
Fermentation process yields for DDR switchgrass (SWG). Due to the unutilized xylose, the fermentation process yields for DDR switchgrass were lower, between 35–42%. SWR DDR (80/120/0.005) appears to be an outlier with a fermentation process yield of only 28%. X/Y/Z represents NaOH conc (g/kg)/residence time (min)/disc-refining gap (in.)

Figure 15 displays the time course data for the DDR switchgrass fermentations, indicating that all available monomeric glucose was consumed much like DDR corn stover and DDR poplar. However, monomeric xylose was not completely consumed, which was not observed for DDR corn stover nor DDR poplar. Figure 16 shows the fermentation process yields for DDR switchgrass. Due to the unutilized xylose, the fermentation process yields for DDR switchgrass were lower, between 35, 42%. SWR DDR (80/120/0.005) appears to be an outlier with a fermentation process yield of only 28%, likely because the fermentations were run in shake flasks which are more difficult to optimize than bioreactors.

## Discussion

### Total process

**Fig. 17:**
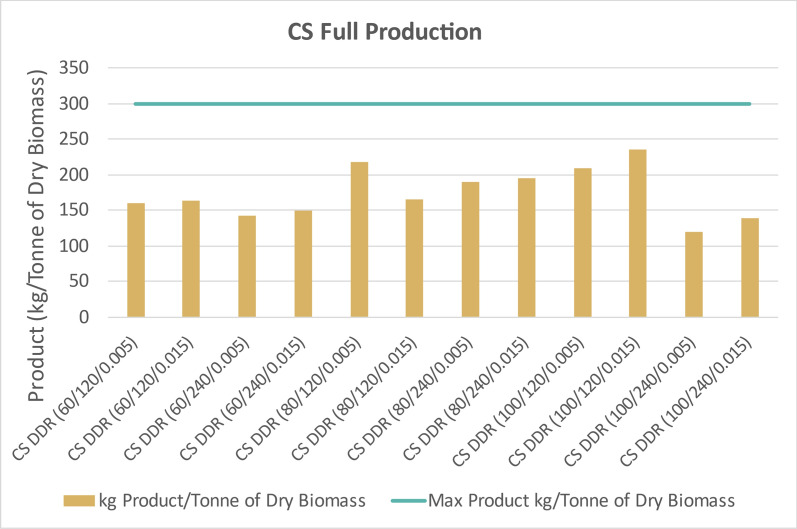
Theoretical integration process yield of fermentation products if individual yields in this study were extrapolated to 1 dry tonne of corn stover (CS). The highest overall yields exceeding 200 kg of product per tonne were achieved for CS DDR (80/120/0.005), CS DDR (100/120/0.005), and CS DDR (100/120,0.015). The theoretical maximum overall product yield from corn stover is 300 kg. X/Y/Z represents NaOH conc (g/kg)/residence time (min)/disc-refining gap (in.)

**Fig. 18:**
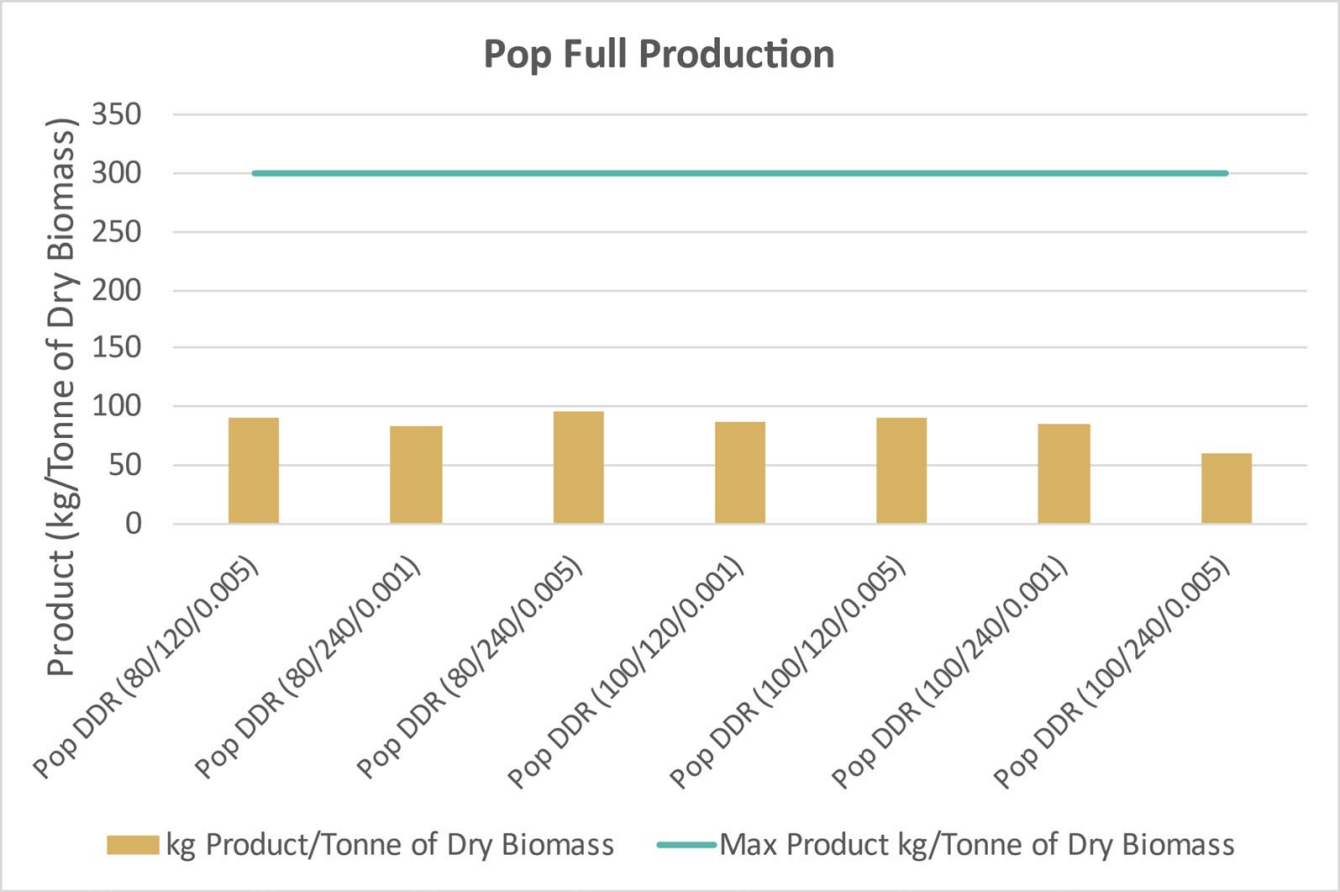
Integrated process yield of fermentation products if individual yields in this study were extrapolated to 1 dry tonne of poplar (Pop). Every DDR condition of poplar yielded less than 100 kg of fermentation products. The theoretical maximum overall product yield from corn stover is 300 kg. X/Y/Z represents NaOH conc (g/kg)/residence time (min)/disc-refining gap (in.)

Figure 17 displays the amounts of fermentation products, 2,3-BDO and acetoin, produced if the individual process yields achieved in this paper were replicated with 1 dry tonne of corn stover. Assuming 100% yields for every individual step, 1 dry tonne of this batch of corn stover would produce 300 kg of fermentation products. Figure 18 indicates how much of the corn stover’s glucan and xylan was ultimately converted to 2,3-BDO and acetoin in relation to the theoretical maximum through deacetylation, disc-refining, enzymatic hydrolysis, and fermentation. The amounts of products produced through the entire process is most strongly dependent on the sodium hydroxide concentration during deacetylation. Residence time and disc-refining gap space did not have a large impact on the overall integrated process yields for DDR corn stover. However, CS DDR (80/120/0.005) may indicate an optimal condition given a similar overall process yield as a higher-severity DDR case using 100 g of sodium hydroxide per 1 kg of dry corn stover.

Figure 18 likewise displays the overall integrated process yields extrapolated to 1 dry tonne of poplar. Assuming 100% yields for every individual step, 1 dry tonne of this batch of poplar would produce 301 kg of products. Overall product yields from poplar were less than 100 kg per tonne of biomass feedstock, half of the yields from corn stover. This was due to poor deacetylation and poor enzymatic hydrolysis performance.

**Fig. 19:**
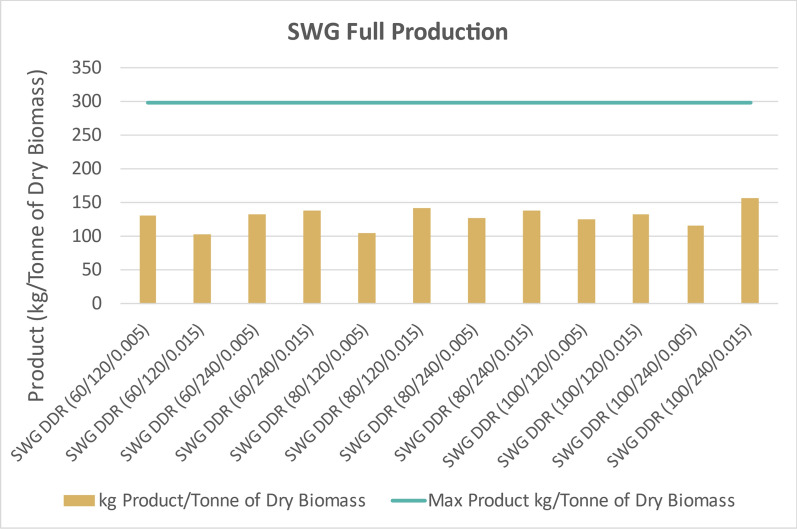
Integrated process yield of fermentation products if individual yields in this study were extrapolated to 1 dry tonne of switchgrass (SWG). Every DDR condition of switchgrass yielded less than 150 kg of fermentation product. This is less than yields from corn stover, but more than poplar. X/Y/Z represents NaOH conc (g/kg)/residence time (min)/disc-refining gap (in.)

Figure 19 displays similar integrated fermentation process yields extrapolated to 1 dry tonne of switchgrass. Assuming 100% yields for every individual step, 1 dry tonne of this batch of switchgrass would produce 299 kg of products. Switchgrass process yields were less than 150 kg of product, which was less than corn stover but more than poplar. The deacetylation and enzymatic hydrolysis processes were less effective with switchgrass than corn stover but superior to poplar. Switchgrass fermentation also incurred penalties from unutilized xylose.

The ability to convert cellulose and hemicellulose into monomeric glucose and xylose post-DDR is the determining factor for overall process yields. The corn stover feedstock was the least recalcitrant of the feedstocks and therefore had the superior process yields. Nearly all of the sugars were converted into 2,3-BDO from the corn stover even with fermentations run in shake flasks. The assumption could be made that if the fermentations were performed in a bioreactor, with pH controls and monitoring of sugar utilization, the yields would improve. While corn stover had superior performance overall, the switchgrass, also herbaceous, had projected product yields greater than 100 kg/Tonne of dry biomass. The poplar was too recalcitrant for significant conversion. In order to better convert cellulose and hemicellulose within poplar, deacetylation would have required high concentrations of sodium hydroxide than what were performed in this study.

### Techno-economic analysis (TEA)

Key TEA results for all three feedstock cases are summarized in Figs. 21-23. These results are based on the financial and cost parameters inherent to nth-plant projections applied in the framework of the sugar TEA model. Because the fermentation was performed in shake flasks and not in a bioreactor where experimental conditions are carefully monitored nor fed batch fermentation possible, the decision was made to report TEA results for the sugars produced in this project.

**Fig. 20:**
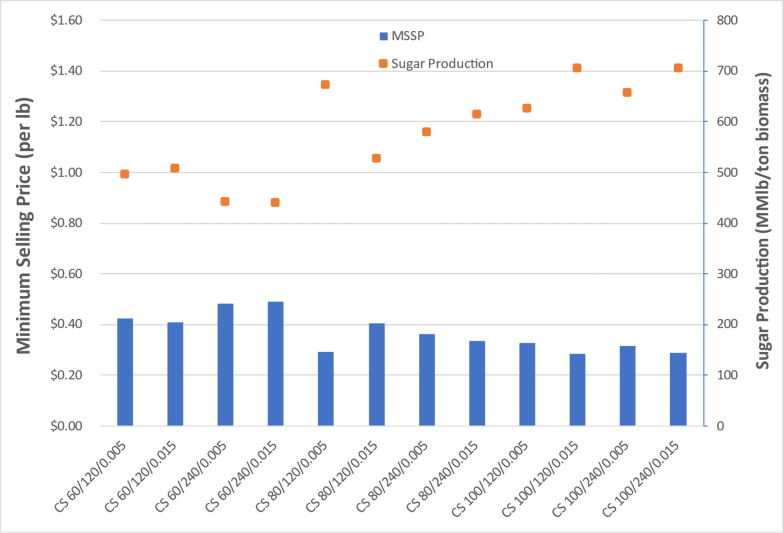
TEA sugar model of DDR corn stover processing through enzymatic hydrolysis. MSSP for corn stover achieved optimal results below $0.30/lb. TEA model results for MSSPs followed similar trends to overall sugar production yields

For corn stover cases, displayed in Fig. 20, the modeled sugar yield ranges from 610 to 976 lb monomeric sugars per dry ton corn stover, corresponding to minimum sugar selling prices (MSSPs) ranging from $0.29/lb to $0.49/lb. The lowest MSSP was $0.29/lb corresponding to the pretreatment condition was using 100 g sodium hydroxide per kg of dry corn stover, 240 min residence time for deacetylation reaction, and 0.015-in. gap space for disc-refining, although the MSSP at a lower sodium hydroxide loading of 80 g/kg with 120 min residence time and 0.005-in. gap space was also seen to be nearly as favorable. For sodium hydroxide loading, as also indicated in the experimental results above 80–100 g of sodium hydroxide per kg corn stover resulted in higher yields than those from 60 g sodium hydroxide. Sugar yields and costs are generally more comparable between 80 and 100 g sodium hydroxide per kg dry corn stover, which implies optimal pretreatment conditions exist within this range of sodium hydroxide loading (although recognizing that sodium hydroxide also carries high carbon intensity burdens, the 80 g/kg loading case my ultimately be more optimal when also considering carbon intensity of the system, which was beyond the scope of this study) [16]. One exception was CS DDR 80/120/0.015, shown in Fig. 20 to incur a higher MSSP and lower sugar process yield than the other cases in the 80–100 g/kg sodium hydroxide loading range. The impacts to sugar yield from varying the residence time of the deacetylation reaction or disc-refining gap space are not clear or consistent winner.

**Fig. 21:**
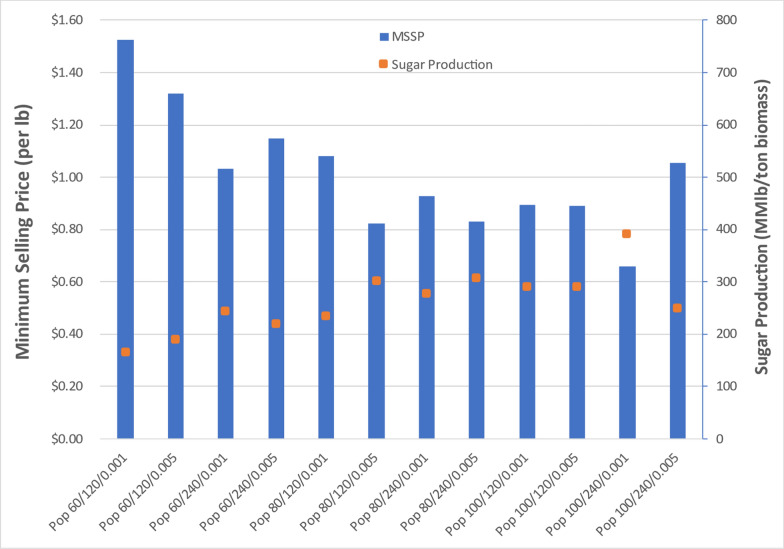
TEA sugar model of DDR poplar processing through enzymatic hydrolysis. The most optimal MSSP for poplar was $0.66/lb, higher than maximum for corn stover. Poor deacetylation and enzymatic hydrolysis performance resulted in low sugar production yields and thus high sugar costs with poplar

For poplar cases, displayed in Fig. 21, sugar yields are lower than achieved from corn stover, ranging from 228 to 539 lb/ton poplar, translating to substantially higher MSSPs ranging from $0.66/lb to $1.52/lb. Both total capital investment and total operating expenses are less favorable than corn stover or switchgrass feedstocks. The best pretreatment condition was seen at 100 g sodium hydroxide per kg of dry poplar, 240 min residence time for deacetylation reaction and 0.005-in. gap space for disc-refining. Even for this case, the sugar yield was 539 lb/ton poplar, lower than most of the cases with corn stover. Sodium hydroxide loading greater than 100 g sodium hydroxide per kg of dry poplar may be needed to achieve more favorable sugar yields with the more highly recalcitrant poplar feedstock.

**Fig. 22:**
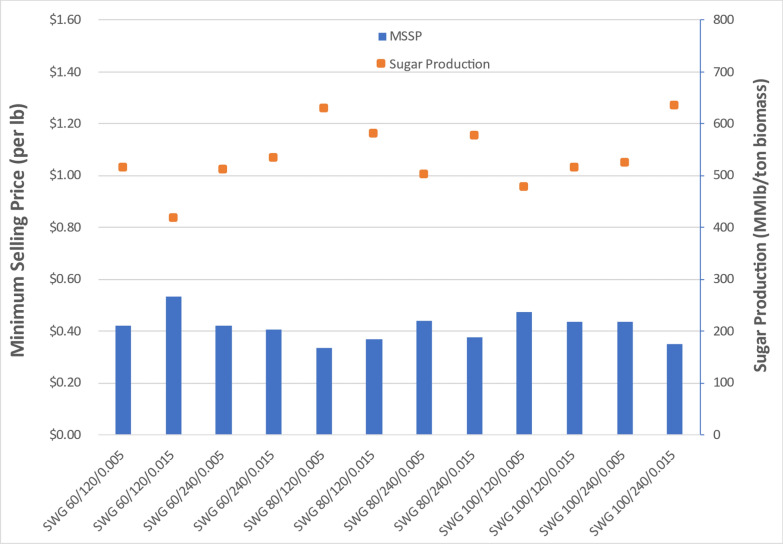
TEA sugar model of DDR switchgrass processing through enzymatic hydrolysis. The most optimal MSSP for switchgrass was $0.33/lb, higher than the optimal cases for corn stover, but lower than for poplar

For switchgrass cases, displayed in Fig. 22, sugar yield ranges from 578 to 877 lb/ton switchgrass with corresponding sugar costs (MSSPs) ranging from $0.33/lb to $0.53/lb. The lowest MSSP is 33 cents/lb corresponding to the optimal pretreatment condition at 80 g sodium hydroxide per kg of switchgrass, 120 min residence time and 0.005-in. gap space. Similar to corn stover cases, a sodium hydroxide loading of at least 80 g/kg appears to be required for switchgrass feedstocks. When compared to corn stover, switchgrass is slightly more recalcitrant, translating to lower sugar yields and higher MSSPs, or may otherwise require more severe pretreatment conditions.

The TEA followed similar trends that were displayed in project productions for each feedstock. The most important factor was the ability to convert cellulose and hemicellulose into monomeric, fermentable sugars. Corn stover was the most favorable feedstock when deacetylated with 80 g of sodium hydroxide or greater.

## Conclusion

The purpose of this study was to compare corn stover, a feedstock that has been extensively studied through these processes versus other promising feedstocks for lignocellulosic biomass conversion to sugars or downstream fermentation intermediates. Additionally, varying the conditions of deacetylation and disc-refining identified optimal operating conditions. Corn stover was observed to be the optimal feedstock based on the conditions investigated. Deacetylation of corn stover was effective at removing more lignin, without significantly solubilizing the cellulose or hemicellulose. Switchgrass lignin was not removed as extensively as corn stover and had more hemicellulose solubilization during deacetylation. Poplar was seen to be too recalcitrant for effective deacetylation under the conditions employed, leading to higher lignin retention which impeded yields through downstream operations. DDR proved to yield minimum amounts of inhibitors through enzymatic hydrolysis and fermentation without degrading glucose and xylose in all cases. The most important and variable operation for DDR across all three feedstocks was the enzymatic hydrolysis, which was reflected in the TEA model and overall production yield results. Poor enzymatic hydrolysis of poplar achieved the lowest yields and highest costs. Poplar’s poor enzymatic hydrolysis performance was the likely result of lignin not being sufficiently removed during deacetylation and potential incompatibilities in the enzyme cocktail not being optimized for hardwood. Enzymatic hydrolysis on switchgrass feedstock was more effective than poplar, but less than corn stover, likely driven by similar challenges in deacetylation efficacy and optimal enzyme compatibility versus corn stover. Fermentations for all three feedstocks had high glucose and xylose consumption with only a small amount of unconverted xylose remaining with switchgrass.

With the most optimal TEA results observed with corn stover, it was clear that deacetylation with 60 g sodium hydroxide loading per kg of dry biomass was not adequate to sufficiently remove lignin and ultimately achieve good hydrolysis sugar yields. However, sodium hydroxide concentration in the range of 80–100 g per kg of dry corn stover achieved more optimal MSSP ranging from $0.29–$0.40. The residence time and gap space during disc-refining did not have as substantial effect on MSSP relative to sodium hydroxide for conversion of all biomass feedstock types.

## Methods

### Biomass feedstock sourcing and preprocessing

Corn stover, poplar, and switchgrass were provided by Idaho National Laboratory (INL). Corn stover was harvest on October 23, 2018, in Iowa. Corn stover was received by INL on December 13, 2018, and stored in bale stacks 6 high and 3 wide. Corn stover was sampled for milling and processing on June 2, 2020. Corn stover was processed through a Vermeer BG480 tub grinder through 2-in. screen. 7600 lb of processed corn stover was shipped to NREL across 29 supersacks. Poplar was harvest on October 20, 2020, in Oregon. Poplar was received by INL on October 22, 2020. Poplar was sampled for milling and preprocessing on January 19, 2022. Poplar was processed through a Forest Concepts Crumbler M24 and sieved through a Forest Concepts Orbital Screen 2448-3 (1/4-in. screen on top and 3/32-in. screen on bottom). 6000lb of processed poplar was shipped to NREL across 15 supersacks. Switchgrass was a mixture of two different harvests at different locations. The first location was from Virginia and harvested on September 1, 2018 and received by INL on July 25, 2019. The second location was from Nebraska with an unknown harvest date and received by INL on March 3, 2020. Switchgrass was stored in bale mixture in bale stacks 6 high and 3 wide. Switchgrass was sampled for milling and processing on July 19, 2022. Switchgrass was processed through a Vermeer BG480 tub grinder through 3/4-in. screen. 9500 lb of processed switchgrass was shipped to NREL across 19 supersacks.

### Biomass feedstock compositional analysis

**Fig. 23:**
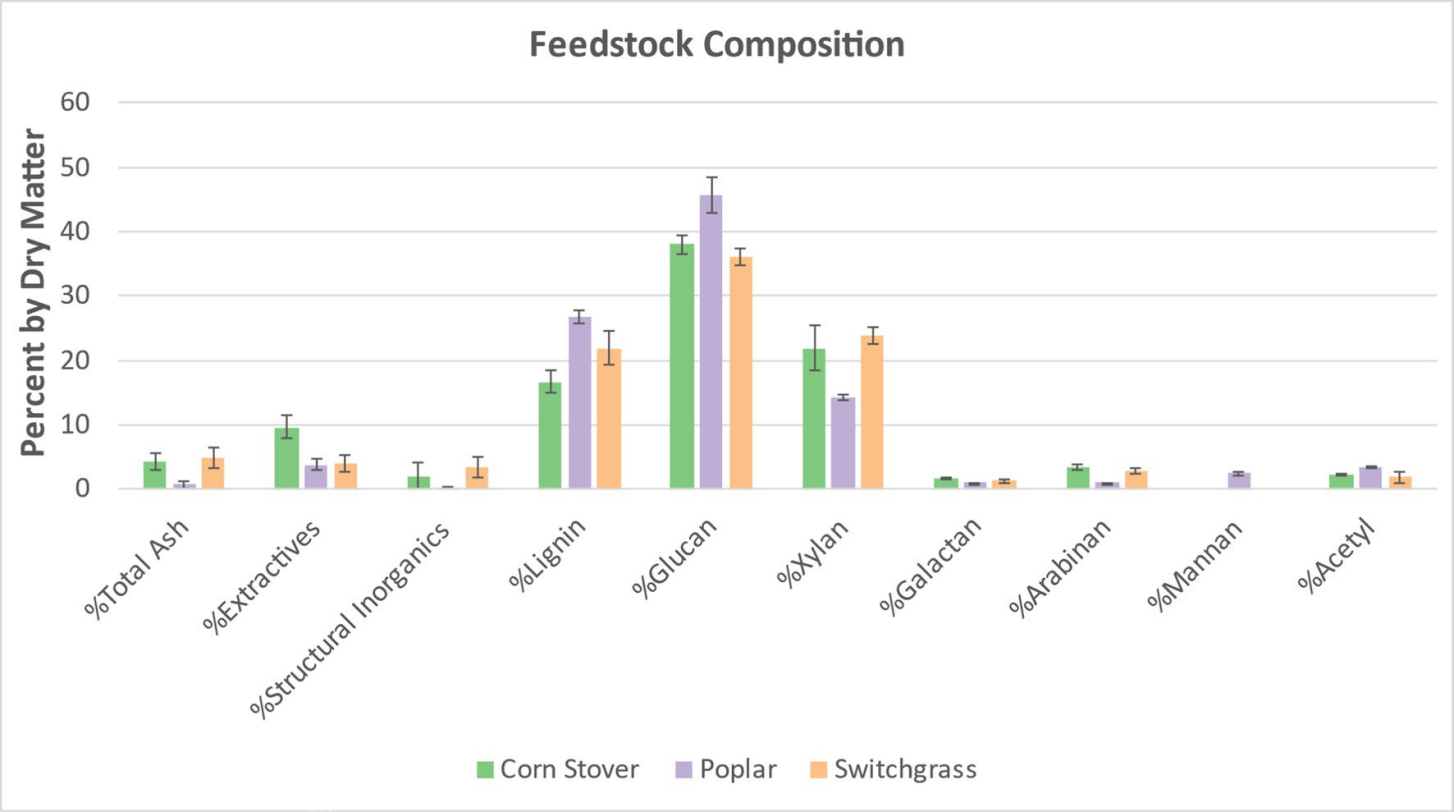
Compositional analysis of corn stover, poplar, and switchgrass with 95% confidence intervals for randomly sampled supersacks

Three-six random supersacks of each feedstock were selected and randomly sampled at six different locations varying in depth, width, and height. Compositional analysis was performed using NREL’s Laboratory Analytical Procedure (LAP), “Determination of Structural Carbohydrates and Lignin in Biomass” [17]. Because the pretreatments were performed at pilot scale for which multiple supersacks were utilized, the compositions for each feedstock were averaged. The average compositions with their 95% confidence intervals are illustrated in Fig. 23.

### Deacetylation

The DDR pretreatment processes used in this project consisted of three steps: deacetylation, solid–liquid separation via screw press, and disc-refining. The deacetylation was completed by soaking approximately 5 kg (dry basis) of biomass feedstock in a solution of sodium hydroxide of varying concentrations inside a custom built jacketed 90-L paddle reactor at 92 °C. The sodium hydroxide concentration used for deacetylation ranged from 60 to 100 g of sodium hydroxide per kg of dry biomass. Water was added to bring the total mass of the slurry to 45 kg. The residence time within the paddle reactor ranged from 120 to 240 min. The same sodium hydroxide concentrations and residence times were used for all three feedstocks for direct comparison. The resulting slurry of biomass and sodium hydroxide solution was then removed and fed through a screw press (VincentCorp, Tampa, FL). The deacetylated slurry was fed into the screw press via continuous feed through the top grate. Inside the screw press an air-pressurized sliding piston pushes the slurry through a dewatering press. The outside cone of the press has a screen which allows the lignin-rich deacetylated liquor, black liquor, to escape and be collected. The pressed deacetylated solids fraction collects and drops out of the bottom of the press into a collection container. The solids fraction collected ranged from 30 to 45% solids. A homogenized sample of each solids fractions were collected and submitted for compositional analysis, described in “Analytical” section. The black liquor samples were also collected and analyzed to quantify how much lignin, acetate, and carbohydrates from cellulose and hemicellulose were removed during deacetylation, described in “Analytical” section.

### Disc-refining

The entire solids fraction collected was disc-refined with a 12-in. two-plate disc-refiner (Sprout-Waldron Operation, Koppers Company, Inc., Muncy, PA). The two refining plates are first secured. Once secured the motor is turned on which spins one disc while the second disc remains stationary. The gap space of the discs is adjusted to a gap less than 0.00 1in, the discs’ closest position. From there, the discs’ are adjusted to the desired gap space. The gap space for corn stover and switchgrass ranged from 0.005–0.015 in. and 0.001–0.005 in. for poplar. Poplar needed a smaller gap space between discs because the deacetylated poplar fell through the disc-refiner at 0.015 in. without sheering. Pressurized water was applied to the surface of the discs to help remove material. The pressed deacetylated solids were hand fed through the top grate of the feed hopper compression screw which conveys the material to the grinding discs. The material was sheered by the grinding discs and was dropped below the refiner to the collection vessel. The disc-refined material is then put through the screw press to dewater the collected material, as described in “Deacetylation” section. Disc-refining was used to help breakdown the cellulose and hemicellulose by sheering and refining the material into smaller particle sizes for better digestion during enzymatic hydrolysis [7–9]. The gap space between discs were varied to determine the impact on enzymatic hydrolysis conversion yields. A full table of DDR pretreatments is displayed in Table 1. A homogenized sample of the disc-refined solids was collected and submitted for compositional analysis as described in “Analytical” section.

**Table 1:** Table of DDR pretreatment conditions

Condition #	NaOH loading (g/kg dry biomass)	Residence time (min)	Disc-refine gap CS & SWG (in.)	Disc-refine gap pop (in.)
1	60	120	0.005	0.001
2	60	120	0.015	0.005
3	60	240	0.005	0.001
4	60	240	0.015	0.005
5	80	120	0.005	0.001
6	80	120	0.015	0.005
7	80	240	0.005	0.001
8	80	240	0.015	0.005
9	100	120	0.005	0.001
10	100	120	0.015	0.005
11	100	240	0.005	0.001
12	100	240	0.015	0.005

Table of DDR pretreatment conditions. The sodium hydroxide concentration ranged from 60 to 100 g per kg of dry biomass and the residence time ranged from 120 to 240 min for deacetylation. Corn stover and switchgrass had the same gap space, 0.005–0.015 in. between discs in the disc-refiner while poplar used a smaller gap space, 0.001–0.005 in. between discs due to particle size after deacetylation

### Enzymatic hydrolysis

Enzymatic hydrolysis was performed on subsamples of each DDR solids fraction. Measuring the digestibility of the deacetylated disc-refined material was necessary to understand the overall impact sodium hydroxide concentrations, residence times, and gap space during disc-refining have on each feedstock during enzymatic hydrolysis. Enzymatic hydrolysis was performed in a laboratory scale roller bottle apparatus. This enzymatic hydrolysis procedure was similar to NREL’s LAP, “Low Solids Enzymatic Saccharification of Lignocellulosic Biomass” [18]. Changes were made to the method to allow for fermentation at a later date and to improve the overall economics. The enzymatic hydrolysis was done at 20% solids loading opposed to the 1% solids loading in the referenced procedure. Sodium azide and citric acid buffer were omitted from the enzymatic hydrolysis to allow for fermentations downstream. The pH was adjusted to 5.2 prior to the addition of the cellulase and hemicellulase enzyme cocktail. The pH was not controlled or monitored once the enzyme cocktail was introduced.

Novozymes Cellic^®^ CTec3 (CTec3) and Novozymes Cellic^®^ HTec3 (HTec3) were the cellulase and hemicellulase used during enzymatic hydrolysis. The total enzyme loading for enzymatic hydrolysis was 15 mg protein/g glucan. A blend of 80:20 (CTec3 vol: HTec3 vol) was used for all the enzymatic hydrolyses. CTec3 is an enzyme cocktail designed to break down cellulose to produce monomeric glucose while HTec3 is designed to breakdown hemicellulose to produce monomeric xylose.

1 mL samples were removed for each roller bottle on days 1, 2, 3, and 5 then submitted for HPLC analysis, described in “Analytical” section. The completed enzymatic hydrolysis slurries were submitted analyses described in “Analytical” section. Day 0 concentrations and percent solids were calculated from the pretreated slurry analytical data.

### Fermentation

The fermentations were conducted on the same 36 hydrolysates generated by enzymatic hydrolysis after solid–liquid separation to remove remaining insoluble solids. Fermentations were conducted in 125-mL unbaffled shake flasks. All fermentations were conducted in duplicate when sufficient hydrolysate was available. The filtered hydrolysates from the different samples contained a wide range of compound concentrations.

Due to the large number of hydrolysates and the desire to perform the fermentation tests in duplicate, it was not possible to logistically conduct all the shake-flask fermentations in a single campaign. Therefore, four separate campaigns were conducted. In each campaign, each hydrolysate was run in single flasks.

A single inoculum flask was prepared for each campaign, which was subdivided and used to inoculate all flasks in each fermentation campaign once the cell growth in the inoculum flask achieved an optical density (OD) of > 5.0. In general, 32 g of filtered hydrolysate and 8 g of combined inoculum and media (rich media [RM] containing 20 g/L glucose, 10 g/L yeast extract, and 2 g/L KH2PO4) were combined to achieve a target starting OD of 0.5 in the fermentation flasks. The starting pH prior to inoculation was 6.0 (hydrolysate adjusted with ammonium hydroxide) and was not controlled or adjusted during the shake-flask fermentations. Once inoculated, each flask was sampled at 0, 6, 12, 24, 48, and 72 h. A typical arrangement for the shake flasks for a single campaign inside of a temperature-controlled orbital shaker (temperate was controlled at 30 °C).

Time-point samples taken during the fermentation were centrifuged and supernatants filtered through a 0.2-μm syringe filter before being placed in high-pressure liquid chromatography (HPLC) vials. The samples were immediately centrifuged, and supernatants filtered through a 0.2 μm syringe and submitted for HPLC analysis as described in “Analytical” section.

The YC-1 strain of *Z. Mobilis* in dictated by the amount dissolved oxygen during agitation of the fermentation [14]. Both acetoin and 2,3-BDO have the same theoretical production yields and both can be upgraded to fuel intermediates. Therefore, the production of acetoin and 2,3-BDO were summed as overall product.

### Analytical

The total solids analysis of the deacetylated feedstock biomass, black liquors, enzymatic hydrolysis slurries and liquors, and fermentation liquors were performed using NREL’s LAP, “Total Solids in Biomass and Total Dissolved Solids in Liquid Process Samples” [19]. Due to the solids and black liquor being caustic, the weighing procedure was changed to using a plastic pan to prevent the sodium hydroxide from reacting with a metal pan. The black liquor samples were also dried in a 40 °C vacuum oven for three days instead of 12 h in a 105 °C oven. Total solids of whole slurries and liquid fractions were used to calculate insoluble solids fractions which were used to calculate procedural yields.

The chemical composition analysis of the black liquor, enzymatic hydrolysis liquors, and fermentation liquors followed NREL’s (LAP), “Sugars, Byproducts, and Degradation Products in Liquid Fraction Process Samples” [20]. All standard high-performance liquid chromatography (HPLC) methods were ran on Agilent 1100/1200/1260 Infinity II/1290 Infinity II system. Due to the high lignin and sodium concentrations of black liquor, some of the procedure was modified. Instead of using a HPLC for quantification of sugars, a Dionex ICS-5000+ converted for high-performance anion-exchange chromatography with pulsed amperometric detection (HPAE-PAD). The eluent was 0.001 M KOH, 50 µl injection, a flow rate of 1.5 mL/min, a Dionex SA-10 column at 45 °C, and a detector temperature of 35 °C. The organic acids and byproducts were quantified using the method in the NREL LAP, but due to the acidic eluent, the black liquor was acidified to 4% sulfuric acid and filtered to remove the precipitated lignin. The method was also modified to quantify 2,3-BDO and acetoin for quantification of fermentation products.

The chemical composition of the solids after deacetylation, disc-refining, and enzymatic hydrolysis were performed using NREL’s LAP, “Determination of Structural Carbohydrates and Lignin in Biomass” [17]. Because the solids fraction had residual sodium hydroxide and black liquor byproducts present, the solids fraction was washed with water to a neutral pH and dried prior to analysis. Similarly, enzymatic hydrolysis solids had residual sugar present and were washed in order to quantify the remaining compositional solids fraction. All HPLC methods were ran on Agilent 1100/1200/1260 Infinity II/1290 Infinity II system.

### Techno-economical analysis

Using a process simulation model based in Aspen Plus V10 with the functionality to reflect deacetylation and mechanical refining or disc-refining, a techno-economic analysis was conducted leveraging NREL’s published biochemical sugar model [21] with modifications for reflect experimental data presented herein and updated to more recent cost and financial parameters [13]. The TEA modeling was performed on all three feedstocks, corn stover, poplar, and switchgrass for all 12 variable conditions, sodium hydroxide concentrations, residence time during deacetylation, and gap space for disc-refining. Due to the fermentations being run in a shake flask setup, which is not representative of batch fermentation kinetics or yields at commercially relevant conditions (as intended to be reflected in the TEA model), the scope of the TEA analysis was limited to sugar production, excluding fermentation or other downstream conversion steps to intermediate/finished fuels. However, trends in resulting minimum sugar selling prices (MSSPs, the selling price for clarified sugars following solid–liquid separation as required to achieve a 10% rate of return at zero net present value) can be extrapolated to comparative economics that would be expected for downstream fuel production. All methods and assumptions for TEA modeling are consistent with previously published practices [13].

## Data Availability

Data will be made public on NREL’s public database once approved.

## Abbreviations

NREL: National Renewable Energy Laboratory
HMF: Hydroxymethylfurfural
DDR: Deacetylation disc-refining
TEA: Techno-economic analysis
DMR: Deacetylation mechanical refining
2,3-BDO: 2,3-Butanediol
MSSP: Minimum selling sugar prices
INL: Idaho National Laboratory
NaOH: Sodium hydroxide
CS: Corn stover
Pop: Poplar
SWG: Switchgrass
CRADA: Cooperative Research and Development Agreement
